# A standardised approach for determining heat tolerance in cotton using triphenyl tetrazolium chloride

**DOI:** 10.1038/s41598-021-84798-2

**Published:** 2021-03-08

**Authors:** Susan Y. Jaconis, Alan J. E. Thompson, Shanna L. Smith, Chiara Trimarchi, Nicola S. Cottee, Michael P. Bange, Warren C. Conaty

**Affiliations:** 1 CSIRO Agriculture and Food, Locked Bag 59, Narrabri, NSW 2390 Australia; 2 USA Dry Pea and Lentil Council, 2780 West Pullman Road, Moscow, ID 83843-4024 USA; 3 NSW Environment Protection Authority, 4 Parramatta Square, 12 Darcy Street, Parramatta, NSW 2124 Australia; 4 GRDC (North), 214 Herries St, Toowoomba, QLD 4350 Australia

**Keywords:** Plant breeding, Heat

## Abstract

Improving the heat tolerance of cotton is a major concern for breeding programs. To address this need, a fast and effect way of quantifying thermotolerant phenotypes is required. Triphenyl tetrazolium chloride (TTC) based enzyme viability testing following high-temperature stress can be used as a vegetative heat tolerance phenotype. This is because when live cells encounter a TTC solution, TTC undergoes a chemical reduction producing a visible, insoluble red product called triphenyl formazan, that can be quantified spectrophotometrically. However, existing TTC based cell viability assays cannot easily be deployed at the scale required in a crop improvement program. In this study, a heat stress assay (HSA) based on the use of TTC enzyme viability testing has been refined and improved for efficiency, reliability, and ease of use through four experiments. Sampling factors that may influence assay results, such as leaf age, plant water status, and short-term cold storage, were also investigated. Experiments conducted in this study have successfully downscaled the assay and identified an optimal sampling regime, enabling measurement of large segregating populations for application in breeding programs. The improved HSA methodology is important as it is proposed that long-term improvements in cotton thermotolerance can be achieved through the concurrent selection of superior phenotypes based on the HSA and yield performance in hot environments. Additionally, a new way of interpreting both heat tolerance and heat resistance was developed, differentiating genotypes that perform well at the time of a heat stress event and those that maintain a similar performance level to a non-stressed control.

## Introduction

High temperature stress is a major limiting factor for crop production, particularly when temperature extremes coincide with critical stages of plant development such as growth, flowering and reproductive development^1^. All plant processes are sensitive to, and can be irreversibly damaged by exposure to high temperatures. In general, high temperatures accelerate senescence, reduce the duration of viable leaf area, and reduce carbon assimilation. High temperatures also affect the thylakoid membranes, reducing the number of chloroplasts per cell^2^. Heat stress is difficult to accurately define as a plant’s response will depend on the duration, rate and severity of the exposure, as well as previous thermal adaptation and phenology at the time of exposure.

**Figure 1:**
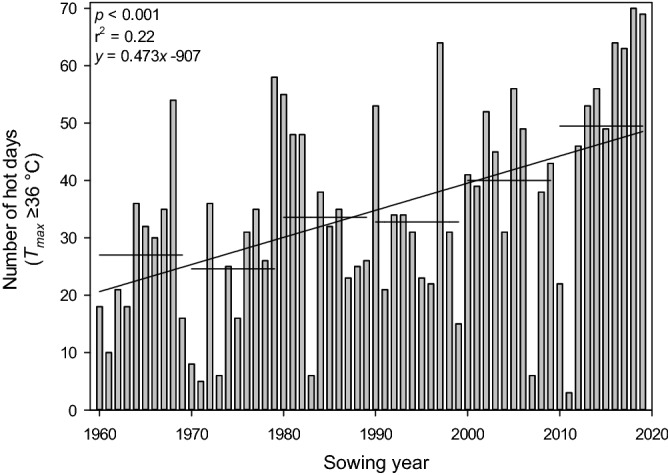
The number of hot days where the maximum temperature reached or exceeded 36 °C in the cotton-growing season at Wee Waa, NSW (15 km west of the Australian Cotton Research Institute, ACRI). Data is between 1960 and 2019 and from Oct. 15 to Apr. 15, representing the duration of the modern Australian cotton industry. Despite the substantial year-to-year variation, the regression line shows a > 4 d increase in the number of hot days for a 10-year period. Horizontal lines represent the decadal average number of heat shock days. Source: CSIRO^8^.

The optimal ambient day/night air temperature for cotton growth and development is approximately 30/22 °C^3^. However, in the semi-arid irrigated and rainfed Australian cotton-growing regions, heat waves routinely expose cotton to temperatures exceeding this optimum, and the number of heat stress days experienced is increasing (Fig. 1). This is critical as the temperature is one of the principal factors affecting cotton growth and development, with negative implications on yield and fibre quality when ambient temperatures are outside of the optimal range^4–6^. The negative effects of heat stress occur when cotton plants can no longer avoid or minimise in vivo heat stress through the regulation of their leaf temperature to the optimum of 28 °C^7^. This high-temperature regulation is primarily achieved through the process of transpiration when air temperatures exceed 28 °C, which results in the reduction of plant temperature through latent heat flux associated with evaporation.

Heat tolerance is a widely studied adaptive mechanism, where exposure to a mild heat stress event induces tolerance at higher temperatures. This acquired thermotolerance occurs under natural conditions and is a component of field heat tolerance^9^. Current conventional breeding approaches to enhance heat tolerance are limited to yield performance data of genotypes grown under hot environments from multiple growing seasons and locations. However, rapid and reliable early-season screening methods can capture innate varietal differences in heat tolerance. Such screening methods could provide an additional avenue for developing germplasm with increased heat tolerance that can then be introgressed into commercial cotton breeding programs. This is because selections on the basis of yield are subject to long intergenerational timelines and performance results may be confounded by environmental and biotic pressures^10^. Furthermore, selections based solely on yield may not necessarily capture complex physiological traits, like heat tolerance. This is because of inherently lower yield potential and negative associations between yield and heat tolerance, which can be associated with heat-tolerant germplasm, resulting in selection pressure for yield negatively affecting heat-tolerant material.

Exposure of plant cells to heat results in cellular membrane disruptions that are related to temperature-specific phase changes in the membrane lipid bilayer^11^. Heat stress events can affect cellular membranes through the plasmalemma^12^, photosynthetic membranes^13^, and mitochondrial membranes^14^. The effects of a heat stress event on mitochondrial membranes can be assessed through mitochondrial electron transport activity, as measured by a cellular viability assay based on triphenyl tetrazolium chloride (TTC) reduction. This assay measures the reduction of TTC by electrons from the mitochondrial electron transport chain^14^. This reduction of TTC by dehydrogenase complex 1 respiratory enzymes produces tetrazolium formazan, a soluble red product which can be quantified spectrophotometrically^15–17^. The amount of formazan product produced directly relates to mitochondrial electron activity, and is proportional to the amount of oxidative damage to respirational processes^14^. Low spectrophotometric absorbance readings at 530 nm indicate a weak reduction (i.e. less red product) and a high value is indicative of a strong reaction (i.e., more red product)^18,19^. Following an imposed heat stress event, high absorbance readings indicate reduced oxidative damage to the respirational processes which is indicative of higher enzyme and cellular viability due to adaptive heat tolerance capacity. Alternatively, impaired enzymatic activity is represented by low absorbance values from low levels of formazan being reduced by the dehydrogenase complex^16,18,19^.

The use of tetrazolium chloride to identify temperature tolerance in plant material has been previously established in numerous crop species^9,18,20–22^, including cotton^15,23,24^. Specifically, Chen et al^18^ conclude that viable cells' ability to reduce tetrazolium chloride can be used as a measure of heat tolerance for both experimental use and genotype selection. In cotton, a study of six cultivars identified heat-tolerant cultivars as those producing more formazan over time in the stress treatment when compared to a control treatment^24^. Studies by Cottee et al^15^ further support these findings, whereby exposure of leaf tissue samples to elevated (40 °C or 45 °C for discerning differences in cotton cultivars) or optimal temperatures in a water bath before adding a TTC solution was used to assess enzymatic performance and the effects of elevated temperature stress. This is further supported by the observation that in germplasm with contrasting levels of heat stress tolerance the regulation of genes associated with the dehydrogenase complex differ^25^, and these differences relate to cultivar performance using the HSA^15^.

While the use of TTC has often been used to simply show cell viability, the physiology underpinning the biological plant processes leading to the production of red formazan in the presence of TTC^26^ makes this chemical useful in screening for stress tolerance and resistance in plant cells. Cottee et al^15^ employed a multi-level approach to the screening of germplasm for heat tolerance by investigating cellular responses to thermal stress (i.e., membrane integrity measured via relative electrical conductivity and enzyme viability measured via the TTC assay) and relating these to physiological measures (i.e., photosynthesis, stomatal conductance, transpiration rate, chlorophyll fluorescence, and electron transport rate) as well as yield, fruit retention, and boll number. Cottee et al^15^ found that the differences at each level reinforced the observed heat tolerance at higher levels of plant function. Therefore, while it is recognized that the TTC assay only targets one mechanism of heat tolerance and provides an indication of the whole plant or crop level heat tolerance, the underlying mechanisms of cultivar specific heat tolerance can be represented in the TTC assay. Thus, the assay is one useful method for identifying and developing heat-tolerant germplasm^15,18^.

The purpose of this study was to advance the various methods using TTC based cellular viability tests^14–16,23^, with the aim of developing a standard methodology for using TTC as a large-scale screening method for identifying and differentiating cotton genotypes with vegetative heat-tolerance. This was achieved by refining the methods used by Cottee et al^15^, providing clarity to the procedure through seven experiments that address possible variations in the methodology. The changes in the methodology were made in the context of specific speed, scale and analysis requirements to be effectively employed in large-scale, field-grown, germplasm enhancement program. The aim of this study is to develop an optimal methodology for the practical application of the HSA to breeding material. It was hypothesised that the size of the leaf discs, sample size, age of the sampled leaf, water status of the plant, the short-term cold storage for the transportation of samples, the duration that leaf tissue is exposed to TTC allowing the reduction reaction to occur, and the duration that leaf discs are submerged in ethanol for the extraction of the formazan product will all affect the results of the HSA. Additionally, a new way of interpreting the results by considering both heat tolerance and resistance is proposed. This study is important as a concurrent selection program utilising yield performance data from hot environments and rapid and reliable physiologically based heat tolerance screening tools will provide the best long-term gains in heat tolerance. At present, no such screening tool can rapidly and reliably identify cotton germplasm with advantageous physiological responses to high-temperature stress. This study presents the HSA methodology which can be used as a physiologically based screening tool for the development of cotton varieties with high-temperature vegetative thermotolerance.

## Materials and methods

### Location and management

Glasshouse experiments were conducted at the Australian Cotton Research Institute (ACRI), Narrabri, NSW, Australia (30°S, 150°E). Glasshouse temperature was controlled at 32 °C day and 17 °C during the night (1900–0500) by an automated heating and cooling system. Approximately 8 cotton (*Gossypium hirsutum* L.) seeds of the commercial variety Sicot 71^27^ (Cotton Seed Distributors, Wee Waa, Australia) were sown in 9L pots of 250 mm diameter. Released in 2002, Sicot 71 was selected for use in this study as it represents a highly successful, broadly adapted Australian cultivar. It is a full season cultivar with a compact growth habit, good disease resistance and good fibre quality package. At its peak in 2003, it occupied 14% of the Australian cotton industry across all climatic zones, while the transgenic version of this cultivar, Sicot 71BRF, occupied 80% of the total Australian cotton area in the 2010/11 season (B. Ross, CSD, pers. comm., 2020). The use of the cultivar Sicot 71 in this research complies with all relevant institutional, national, and international guidelines and legislation.

Pots were filled with a uniform grey cracking clay soil (Australian soil taxonomy: Grey Vertosol, USDA soil taxonomy: Typic Haplustert) with a clay fraction percentage of 60–65%, pH of 8.0–8.8, and inherently low organic matter and nitrogen content from local ACRI fields^28,29^. Prior to planting, soils in pots were kept saturated with water and a basal fertiliser, 10 g MULTIgro (Incitec Pivot Fertilisers, Melbourne, Australia), was added to the soil surface of each pot and dissolved into the soil via hand-held irrigation. MULTIgro is a multi-purpose garden fertiliser with nutrients N, P, K, S and Ca of 13.1, 4.5, 7.2, 15.4, and 2.4%. The soil surface of each pot was covered with approximately 20 mm of moist sand to assist in reducing surface evaporation and ensure even and rapid plant emergence. Sand was maintained moist through daily surface, hand-held irrigation. Following emergence, seedlings at the three-leaf stage were thinned to the desired rate, detailed below. Plants were provided with non-limiting water supply through daily irrigation, where approximately 1200 ml of water over a 15 min period was supplied at 0900 via drip irrigators to saucers underneath pots. To ensure nutrition did not limit plant growth and function, at first flower pots were fertilised with 2 g of Liquifert K (Incitec Pivot Fertilisers, Melbourne, Australia) and 6.5 g GranAm (Incitec Pivot Fertilisers, Melbourne, Australia) dissolved in 500 ml water. Liquifert K is a soluble potassium chloride-based fertiliser (50% K), and GranAm is a granulated ammonium sulphate fertiliser (20.5% N, 23.9% S). Simultaneously, irrigation drippers were moved to the soil surface, ensuring soil water status remained non-limiting. As required, pests (*Bemisia tabaci* Gennadius, *Tetranychus urticae* Koch, and *Frankliniella occidentalis* Pergande) were controlled with the use of commercial pesticides following a standard commercial practice.

Plants were arranged in a completely randomized design on glasshouse benches. *Experiment 1* was sown on 20 December 2009 with a total of 12 plants, 3 per pot. *Experiment 2* was sown on 30 March 2016 with a total of 16 plants, four per pot. *Experiments 3, 4, 5, and 6* were sown on 26 October 2017 with 48 plants, one per pot. For *Experiment 7*, 13 pots were sown on 12 February 2018 with 52 plants, four per pot. All samples were collected during the squaring and early flowering stages of phenological development.

### Heat stress assay protocol (Original method)

Heat tolerance of cotton plants was measured using a 2,3,5 triphenyl tetrazolium chloride salt (TTC) solution method referred to as the heat stress assay (HSA), as described by Cottee et al^15^. Leaf discs 10 mm in diameter were excised from the interveinal portion of the first fully expanded main stem leaf (FFEL, usually third leaf from the plant apex). These samples were kept cool and immediately transported back to the laboratory in paper bags where they were submerged in 2 ml Millipore water contained in 14 ml vials. These vials were then incubated in water baths for 2 h at 25 °C or 40 °C. The 25 °C treatment is considered the non-stresses control, while the 40 °C is the high-temperature stress treatment, the point at which Cottee et al^15^ found genotypic discrimination in HSA results.

Following incubation in the water bath, the vials were cooled to room temperature. A phosphate buffer solution containing 0.01 M phosphate-buffered saline (0.138 M NaCl; 0.0027 M KCl with TWEEN 20 [0.05% v/v]), pH 7.4, at 25 °C) and 0.8% w/v 2,3,5-triphenyl tetrazolium chloride (TTC) was prepared and 8 mL was added to each vial. The leaf discs were held at − 33 kPa for 15 min in a vacuum desiccator to ensure TTC uptake into the leaf and left to incubate at 25 °C in the dark for 24 h. Discs were triple rinsed with Millipore water, submerged in 2 mL of 95% (v/v) ethanol, and incubated for a further 24 h in the dark. Enzyme viability was determined by spectrophotometric absorbance at 530 nm using 95% ethanol as a reference. Data is presented as absorbance values for samples incubated at 25 °C (Abs_25_) and 40 °C (Abs_40_), as well as relative absorbance (Rel_Abs_), which was calculated as the quotient of Abs_40_ and Abs_25_ (Eq. 1).

Equation (1) relative absorbance (Rel_Abs_) is calculated as the quotient of absorbance values at 530 nm for samples incubated at 40 °C (Abs_40_) and 25 °C (Abs_25_).

1 $$ Rel_{Abs} = \frac{{Abs_{40} }}{{Abs_{25} }} $$

### Statistical analysis

Across all experiment Abs_25_, Abs_40_, and Rel_Abs_ were each analysed separately using a general ANOVA and post hoc Tukey tests to examine differences among the treatments (described below through each *Experiment*) using GenStat 18th Edition (VSN International Ltd., Hemel Hempstead, UK). Analyses were conducted on each of the three absorbance calculations to assess their utility in the determination of the heat stress assay as a screening tool. As noted, where necessary data was transformed to fit the assumptions of normality and heterogeneity required for the analyses.

Unless otherwise stated, statistical analysis was conducted using Analysis of Variance (ANOVA) in Genstat.

### Experiment 1: Scaling assay from 14 ml vials to 1.1 ml ELISA tubes

Previously, the HSA method was based on collecting 10 mm diameter leaf discs and then submerging these discs in 2 ml distilled water contained in 14 ml vials. However, it was hypothesized that the size of the leaf disc could be decreased to enable a higher throughput of samples. This is because scaling the collection of samples to a 1.1 ml ELISA tube would allow samples to to undergo the heat treatment and subsequent sample processing on a 96 well plate platform. Such a platform enables throughput to be increased with the deployment of automated/semi-automated plate dispensers and spectrometric plate readers. This experiment was designed to test that HSA results would be consistent when scaling up the number of samples required to implement the HSA as a useful screening tool. This scaling method involves halving the size of leaf discs collected, from 10 mm diameter (~ 80 mm^2^) to 7 mm diameter (~ 40 mm^2^) and reducing the incubation vessels and volume from 2 ml of water in a 14 ml vial to 1.1 ml water in a 1.1 ml ELISA tube.

The downscaled HSA method was essentially the same as the original method^15^, with the following exceptions. Ten leaf discs, each fitting the rim of 1.1 mL ELISA tubes, were collected from the first fully expanded main stem leaf (FFEL, usually third leaf from the top) of each plant along either side of the midrib and immediately submerged in Millipore water by filling the tube. Leaf discs were created by placing the ELISA tube underneath the leaf and carefully pressing a finger on the top of the leaf to provide pressure that gives a clean cut along the inner circumference of the tube to minimise inter-sample contamination. From each plant, five of the ELISA tubes with leaf discs from one side of the leaf midrib were transferred to a 40 °C water bath for two hours. The remaining five leaf tissue samples from each plant were kept at room temperature of 25 °C for two hours. Once incubated for the required duration, the Millipore water was drained from each ELISA tube and replaced with 800µL of 0.8% TTC solution in P-3563 phosphate-buffered saline with Tween 20 (Sigma Aldrich). All samples were then vacuum infiltrated at − 33 kPa for 15 min to ensure thorough uptake of TTC into the leaf tissue. The leaf tissue soaking in TTC solution was kept in the dark for 22 h at 25 °C. In each leaf disc tube, the TTC solution was then replaced with 750 µL of ethanol (95% v/v) and kept in the dark for 22 h at 25 °C. After the 22-h period, the liquid in each sample ELISA tube was homogenized using a pipette and 150 µL was transferred to 96 well ELISA plates for spectrophotometric readings at 530 nm using a plate reader (Bio-Rad Microplate Reader). Spectrophotometric absorbance was measured for all samples exposed to both the 25 °C and 40 °C water bath treatments. Absorbance values for samples incubated at 25 °C (Abs_25_) and 40 °C (Abs_40_) were calculated by removing the inherent absorbance value of the ethanol and adjusting absorbance values based on the position of the ELISA plate. This was achieved through measurement of absorbance on a ‘blank’ ELISA plate where ELISA wells contained 150µL ethanol.

The HSA was sampled 51 days after sowing (DAS), where the method of the original HSA^15^ was compared with the revised method detailed above. Leaf discs from the same leaf were excised for both HSA methods (original and revised). Leaf discs were excised from the interveinal portion of the FFEL. Three plants per pot were sampled from an experiment with four replicates, totalling 12 leaves, each from different plants. Absorbance values were collected from discs incubated at 25 °C and 40 °C and using bath temperature and HSA assay method (original and revised) as treatment factors ANOVA was conducted to detect differences. Following this, a Wald test was conducted to determine if the slope of the fitted regression line of the original and revised HSA methods differed from that of the 1:1 line. As no difference was observed between the original and the revised methods, all following experiments used the down-scaled revised HSA method.

### Experiment 2: Selecting a sample size

This experiment was designed to use the standard error of sample means (SEM) to assess the variability of HSA results. It was hypothesised that an optimal sampling regime could be determined, where sampling additional plants and leaf discs beyond this optimum will not improve resolution in the data by reducing standard errors. It was also hypothesised that practical limitations associated with sample size might also play a role in identifying this optimum. The HSA was sampled on 56 DAS, where data was expressed as Abs_25_, Abs_40_ and Rel_Abs_. Samples were collected as detailed above in the revised method, where eight leaf discs were collected from the FFEL from 11 plants. The number of leaf discs sampled per leaf and the number of plants sampled were used as treatment factors in the experiment. An ANOVA was conducted to detect differences in absorbance by splitting the data into two replicates of 5 plants, where plant number and disc number were treated as experimental factors.

Further analysis of the data was conducted by calculating the SEM for all plant number and leaf disc number combinations. This was achieved by selecting the appropriate plant number and leaf disc number, making sure calculations were conducted on the data in the order that it was collected. This ensured that the identifier data assigned to plant and leaf number was the same for each SEM calculation; i.e., plant 1 and leaf disc number 1, plant 2 and disc 2 et al*.* were the same for all SEM calculations. SEM was also subjected to ANOVA, where again, data was split into two replicates of five plants, each with eight discs collected. Plant number and leaf disc number were treated as experimental factors. Similar trends and conclusions between the SEM for Abs_25_, Abs_40_, and Rel_Abs_ data were observed (data are not shown), and thus only Abs_40_ data is presented.

### Experiment 3: Leaf age selection

This experiment was designed to determine if leaf age has an effect on the results of the HSA. To test this, it was hypothesized that absorbance values from the main stem leaves one node above or below the FFEL would have no difference to those of the FFEL (i.e., the ideal leaf for sampling). The HSA was carried out 69 DAS as detailed above in the revised method; however, leaf age was used as a treatment. Leaf discs were collected from the ideal leaf (i.e. FFEL, or 12 days from leaf unfolding) as well as from the main stem leaf above it (9 days from unfolding) and 3 main stem nodes below in consecution. This created five leaf age treatments of approximately 9, 12, 16, 19, and 23 days from unfolding. Five replicates for each of the leaf age treatments were selected randomly from individual plants for a total of 25 plants sampled. The Rel_Abs_ was square-root transformed to meet the assumptions of normality, the Abs_25_ required no transformation and the Abs_40_ was log base 10 transformed.

### Experiment 4: Plant water status at sampling

To test the effect of water stress on the HSA and to give guidance on the management of plants to be sampled when using the HSA, *Experiment 4* was conducted. It was hypothesized that if visible water stress has no impact on HSA, no difference in absorbance values among plants with or without water stress would be observed. Half of the potted plants (24/48) were randomly selected to have irrigators removed at 0900 on 74 DAS. The following day at 0900 h, when the plant showed visible signs of water stress (i.e. wilting), 8 water-stressed and 8 non-water stress plants were randomly selected to undergo the HSA. It should be noted that water stressing plants for greater than one day made the sample leaf too brittle for accurate sampling. The level of water stress imposed was assessed on the basis of soil moisture content as well as plant water use; i.e. stomatal conductance. Soil water stress level was assessed by inserting a soil moisture sensor (Delta-T Devices ML3 ThetaProbe Soil Moisture Sensor, Cambridge, UK), into three locations in each of the 16 pots until the probes were completely covered with soil. The mean moisture content in each was calculated as the mean of the three measurements in the pot. Plant water stress was quantified through measurements of stomatal conductance. This was conducted prior to leaf disc sampling using a steady-state diffusion leaf porometer (Delta-T Devices Decagon Leaf Porometer SC-1, Cambridge, UK). Each leaf was individually placed in the sensor, ensuring the midrib was not covered and the clamp was closed until reading was taken. Soil moisture and stomatal conductance analysis did not require transformation. The Rel_Abs_ and Abs_40_ were log base 10 transformed while the Abs_25_ required no transformation to meet the assumptions of normality and heterogeneity.

### Experiment 5: Short term cooled storage for transporting samples prior to conducting HSA

Because sampling location and laboratory facilities are not always in close proximity, especially when collecting leaf tissue from field sites, it was necessary to assess the effect of short term cooled storage on the HSA results. It was hypothesized that there would be a window of time in which leaf tissue samples could be stored without affecting the resulting absorbance values. To test this hypothesis, samples were collected 39 DAS according to the HSA method but included a treatment of time in cooled storage immediately after sampling and prior to being placed in the water bath. Leaf disc samples were left in a 39 × 24 × 28 cm cooler at an average temperature of 15.8 °C (14.6–16.2 °C) for 1, 2, 4, 8, or 16 h. One leaf from 5 randomly selected plants was used for each treatment duration for a total of 25 plants. All data were log base 10 transformed to meet the assumptions for ANOVA analysis.

### Experiment 6: Duration of leaf tissue submersion in TTC

The duration of leaf disc samples in the TTC chemical was assessed to determine an ideal duration of submersion. It was hypothesized that after a specific time, the reaction with the solution would be complete and the absorbance values would remain consistent. The original HSA method employed a 22 h duration of leaf disc exposure to the TTC solution. However, this method was primarily based upon ease of operation with respect to the timing of the laboratory steps, not the reliability of results. Samples were collected 46 DAS. The HSA methods were followed with the inclusion of a treatment varying the time leaf tissue was soaked in the TTC solution. Treatment durations were 1, 2, 4, 7, 20, and 24 h (referred to as Run 1). Five plants were randomly selected for each of the 6 treatments for a total of 30 plants sampled. For ANOVA analysis, log base 10 transformation was necessary for the Rel_Abs_ and Abs_40_ while Abs_25_ was square-root transformed to meet the assumptions of normality and heterogeneity. Because the results of Run 1 showed the gap between 7 and 20 h could have important implications, the experiment was repeated as Run 2 with treatment durations of leaf disc submergence in TTC solution set at 10, 12, 14, 16, 18, and 20 h. This was conducted 56 DAS. An unexpected loss of samples set in the 25 °C water bath for the 20-h treatment resulted in Rel_Abs_ and Abs_25_ analyses to only include the 10, 12, 14, 16, and 18-h data. To meet the assumptions of ANOVA tests, the Abs_40_ was log base 10 transformed.

### Experiment 7: Duration of leaf tissue submersion in ethanol

This experiment was designed to test the ideal duration of ethanol extraction of formazan for spectrophotometric analysis. It was hypothesized that a maximum absorbance level would be detected, followed by the degradation of the red formazan product. The original HSA method employed a 22 h duration of formazan product extraction in ethanol. However, again this method was primarily based upon ease of operation with respect to the timing of the laboratory steps, not the reliability of results. Five replicate plants were randomly selected from separate pots for each of 10 treatment durations for a total of 50 plants. The ethanol treatment durations were 1, 2, 4, 8, 12, 16, 20, and 24 h. All plants were sampled at 0900 36 DAS and all steps were followed according to the HSA except for the ethanol submersion step in which durations varied. To conduct ANOVAs, Rel_Abs_, Abs_25_, and Abs_40_ were all log base 10 transformed to fit the assumptions of the analysis. Additionally, linear regression analysis of Abs_25_ and Abs_40_ with the duration of exposure to ethanol were conducted using SigmaPlot 14.0.

## Results

### Experiment 1: Scaling assay from 14 ml vials to 1.1 ml ELISA tubes

**Figure 2:**
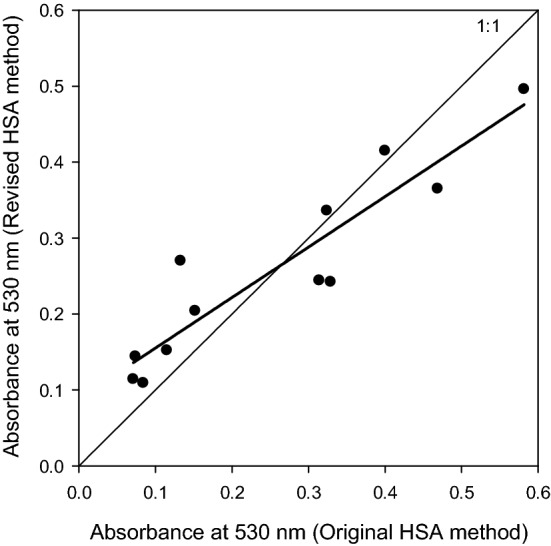
Correlation between absorbance values at 530 nm using the original HSA method published by^15^ and the revised method (as outlined in this manuscript), to enable the screening of larger populations (y = 0.665x + 0.089, r^2^ = 0.86; *p* < 0.001). Note, no statistical difference from the 1:1 line was observed (*p* = 0.08).

ANOVA shows there is no effect of HSA method (*p* = 0.195). While there is an incubation temperature treatment effect (*p* < 0.001), no interaction between incubation temperature and HSA method was observed (*p* = 0.156). Wald test shows no difference in the slope of the 1:1 line (i.e. when slope = 1; *p* = 0.08; Fig. 2), and the slope of the 1:1 line lies within the 95% confidence interval of the fitted regression model (CI = 0.9174551; 1.65949176).

### Experiment 2: Selecting a sample size

**Figure 3:**
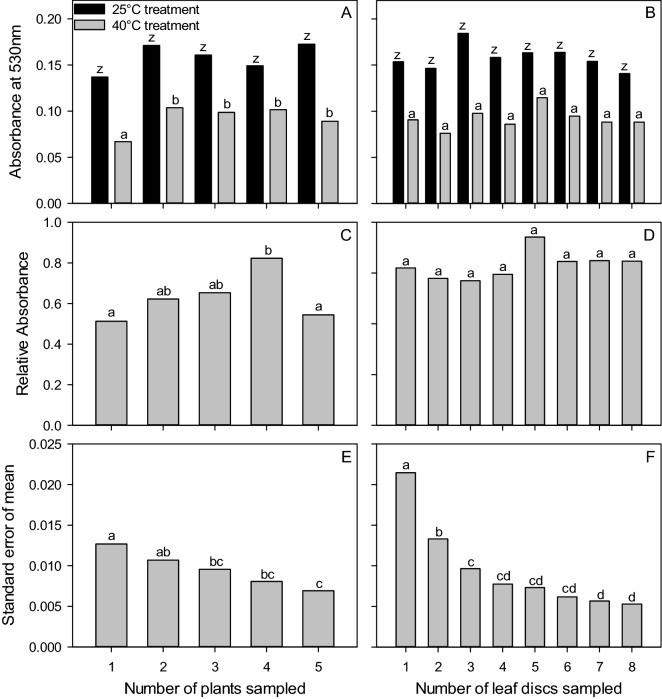
ANOVA and post hoc Tukey test results (depicted as lower-case letters) assessing the difference between absorbance values for the number of plants and leaf discs sampled (**A**–**D**). Absorbance values for 25 °C (Abs_25_, black bars) and 40 °C (Abs_40_, grey bars) treated leaf discs in terms of number of plants sampled (**A**), and number of leaf discs sampled/plant (**B**), relative absorbance values for the number of plants sampled (**C**) and number of leaf discs sampled/plant (**D**). ANOVA and post hoc Tukey test results (depicted as lower-case letters) assessing the difference between standard error of the mean (SEM) of absorbance values at 530 nm for 40 °C treated leaf discs (Abs_40_) with respect to (**E**) the number of plants, and (**F**) the number of leaf discs sampled/plant.

ANOVA showed that Abs_40_ and Rel_Abs_ were altered by the number of plants sampled (*p* < 0.001 and *p* = 0.034, respectively; Fig. 3A,C); where Abs_40_ values observed from one plant differed from those observed by two to five plants. Likewise, was also influenced by the number of plants sampled Absorbance and Rel_Abs_ values remained unaffected by the number of leaf discs sampled (*p* = 0.056 and *p* = 0.914) (Fig. 3B,D), and no plant number-by-leaf disc interaction was observed (*p* = 0.683). ANOVA of the standard error of sample means (SEM), using treatment factors of plant number and leaf disc number revealed differences at both the plant (*p* = 0.002) and leaf disc levels (*p* < 0.001), but no interaction between main effects was observed (*p* = 0.979; Fig. 3E,F). As expected, the SEM was lowest for the maximum number of plants and leaf discs sampled. However, in the case of number of plants, no difference was observed between three to five plants, and in the case of leaf disc number no difference was observed between four to eight discs.

### Experiment 3: Leaf age selection

**Figure 4:**
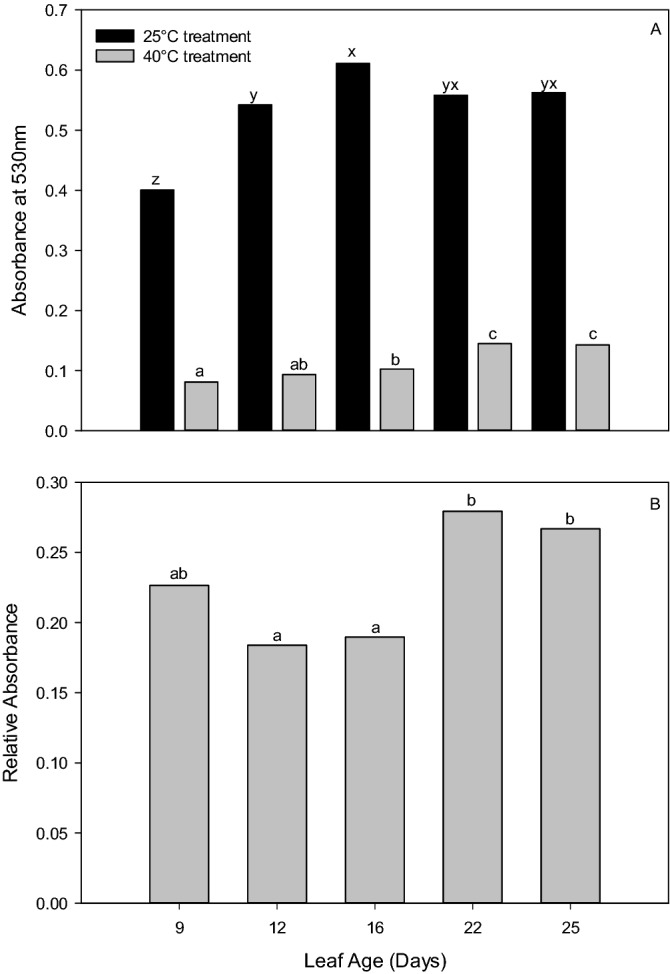
The ANOVA and post hoc Tukey test results (depicted as lower-case letters) of the impact of leaf age on (**A**) absorbance at 530 nm for leaf tissue incubated at 25 °C (Abs25, black bars) and 40 °C (Abs40, grey bars) and (**B**) relative absorbance (Abs40/Abs25). Note, the leaf age of the first fully expanded leaf (FFEL) is 12 d.

Differences in Rel_Abs_, Abs_25_, and Abs_40_ based on leaf age were detected by ANOVA (all analyses, *p* < 0.001; Fig. 4A,B). Consistency in the Rel_Abs_ and Abs_40_ of leaves above and below the FFEL (9 and 16-day old leaves) was detected through the analysis; however, when leaves were older than 16 days, their absorbance values increased (Fig. 4B). Instead, the Abs_25_ of 9- and 16-day old leaves were different from the FFEL (Fig. 4A). However, older leaves at 19 and 23 days old had similar Abs_25_ values to both the FFEL and 16-day old leaves (Fig. 4A).

### Experiment 4: Plant water status at sampling

Visible water stress in plants was due to a 55% water reduction in soil moisture (non-water stressed mean = 34.52%; water-stressed mean = 15.47%; *p* < 0.001). Additionally, stomatal conductance to water vapour was shown to be affected by the water stress, as indicated by a mean stomatal conductance of 493 µmol m^−2^ s^−1^ under non-water stress and 97 µmol m^−2^ s^−1^ under water-stressed conditions (*p* < 0.001).

Although water stress was evident, Rel_Abs_ remained unaffected (*p* = 0.103). However, both Abs_25_ and Abs_40_ showed reductions under water stress conditions (both *p* < 0.001).

### Experiment 5: Short term cooled storage for transporting samples prior to conducting HSA

Leaving leaf tissue samples in cooled storage for transport between one and 16 h had no effect on Rel_Abs_ (mean = 0.6906; *p* = 0.606), Abs_25_ (mean = 0.218; *p* = 0.588), and Abs_40_ (mean = 0.15; *p* = 0.287).

### Experiment 6: Duration of leaf tissue submersion in TTC

**Figure 5:**
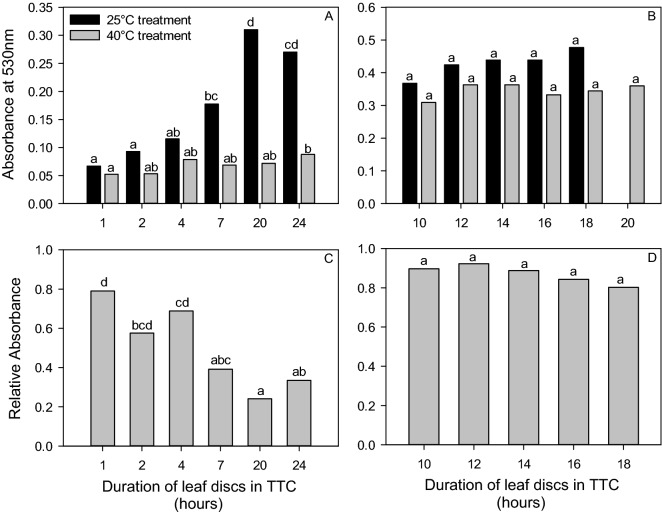
ANOVA and post hoc Tukey test results (depicted as lower-case letters) showing the differences between measurements with respect to duration of leaf disc submersion in TTC. Pane (**A**) and (**B**) show absorbance at 530 nm for leaf tissue incubated at 25 °C (Abs25, black bars) and 40 °C (Abs40, grey bars), where Run 1 with durations from 1 to 24 h is shown in pane (**A**), and Run 2 with durations 10–18 h is shown in Pane (**B**). Relative absorbance values are shown in Panes (**C**) (Run 1 with durations from 1 to 24 h) and (**D**) (Run 2 with durations 10 to 18 h).

The duration of leaf tissue submersion in the TTC solution was shown to affect Rel_Abs_ (*p* < 0.001), Abs_25_ (*p* < 0.001), and Abs_40_ (*p* = 0.026) when ANOVA analysis was conducted on Run 1 based on 1, 2, 4, 7, 20, and 24 h of leaf tissue submersion (Fig. 5A,C). Within each analysis, some similarities were found. For example, Rel_Abs_ values were similar at 7, 20, and 24 h of submersion in TTC as revealed by Tukey test analysis (full results shown Fig. 5C). The Tukey test results also indicated differences among many of the durations for Abs_25_ and are shown in Fig. 5A for clarity. When analysing the Abs_40_ results, similar absorbance values were detected among the 2, 4, 7, and 20 h treatments. These treatment times were also similar to the 1- and 24-h treatments, however, the 1- and 24-h treatments were different from each other (Fig. 5A).

When the treatment durations were narrowed to the 10 to 20-h range for Run 2, differences were no longer observed in the Rel_Abs_ (*p* = 0.683; Fig. 5D), Abs_25_ (*p* = 0.156; Fig. 5B), and Abs_40_ (*p* = 0.431; Fig. 5B).

### Experiment 7: Duration of leaf tissue submersion in ethanol

**Figure 6:**
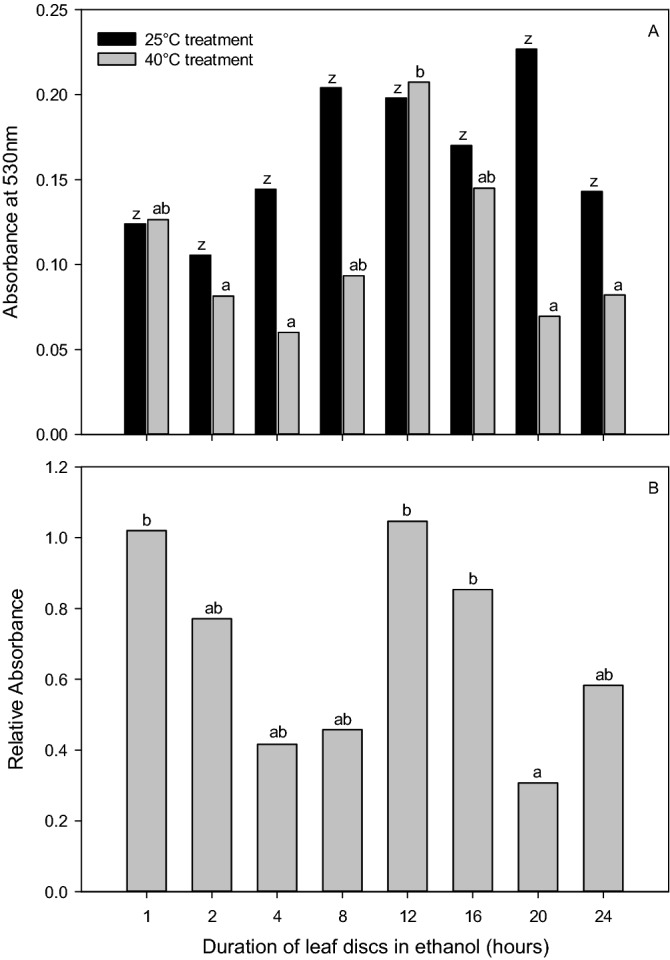
ANOVA and post hoc Tukey test results (depicted as lower-case letters) demonstrating the difference between (**A**) absorbance at 530 nm for leaf tissue incubated at 25 °C (Abs25, black bars) and 40 °C (Abs40, grey bars), and (**B**) Relative absorbance (Abs40/Abs25) with respect to duration of leaf disc submersion in ethanol, from 1 to 24 h.

ANOVA analysis revealed that Abs_40_ and Rel_Abs_ were affected by the duration leaf tissue is exposed to ethanol (*p* = 0.002 for both Abs_40_ and Rel_Abs_). The highest absorbance values were recorded at 12 h for both measurements (Fig. 6). In contrast, the duration leaf tissue was exposed to ethanol did not alter Abs_25_ results (*p* = 0.093). Despite the lack of an effect, similar trends in Abs_25_ and Abs_40_ results were observed. Quadratic relationships were observed when Abs_25_ and Abs_40_ were regressed with the duration leaf tissue was exposed to ethanol. These relationships saw a peak in absorbance at 13.3 ± 4.2 for Abs_25_ (*y* = -0.0006*x*^2^ + 0.0157*x* + 0.0715; *p* < 0.001, r^2^ = 0.39) and 13.1 ± 4.1 h for Abs_40_ (*y* =  − 0.0008*x*^2^ + 0.0213*x* + 0.0956; *p* < 0.001; r^2^ = 0.36).

## Discussion

A screening method must meet the specific speed, scale and analysis requirements to be effectively employed in large-scale, field-grown, germplasm enhancement programs. As such, the following discussion is primarily focused on these requirements and the improved the efficiency, reliability, and ease of use of the HSA methodology.

In this study, we identified that the original methodology by Cottee et al^15^ using 14 ml vials could be downsized to 1.1 ml ELISA tubes. This is an essential improvement on this methodology as it enables the collection and analysis of larger sample sizes or screening of more populations. The smaller size of the leaf disc is easier and faster to collect. Furthermore, it increases throughput in processing as samples can be deployed on a 96 well ELISA plate platform, enabling the use of automated pipettes and plate readers. The ability to scale the sampling procedure is essential for the screening of large numbers of genotypes, which is required in a germplasm enhancement or breeding program. However, with the ability to collect more samples, it is essential to select the appropriate sample size. The sample size must be balanced among the number of genotypes of interest, the number of plant replicates (from which the FFEL will be sampled), and the number of leaf discs collected per plant.

This study showed that the number of plants sampled has a more substantial effect on the measured absorbance than the number of leaf discs sampled. However, the SEM calculated across leaf disc numbers was higher than those observed across the number of plants sampled. This observation, along with SEM structure associated with specified sampling regimes, is essential as reductions in SEM may be critical in determining genotype differences. We determined that when sampling up to eight discs per plant from five plants, there was no improvement in SEM (decreased SEM) in sampling more than 4 leaf discs. Thus, the optimum sampling strategy was found to be four discs from three plants per experimental unit. This sampling regime corresponds with the lowest total number of leaf discs sampled (n = 12), as well as a SEM statistically similar to the smallest observed SEM. However, if collecting a total of 12 leaf discs is not practical for a given experiment the sampling strategy of 3 discs from three plants (n = 9), or as less desirable alternative 3 discs for 2 plants (n = 6), can be employed. A sampling strategy that samples less plants and leaf discs than this would not be recommended, as the observed SEM rises to levels that may impact the ability to resolve genotype differences.

The FFEL is regularly sampled in physiological studies. It is where maximum photosynthetic rates are observed and is used as a representative of the sunlit portion of the canopy^30^. However, from a practical standpoint when collecting tissue samples, the FFEL may not always be available for sampling due to complications such as herbivory or developmental issues, as well as differences in the judgment of the person sampling as to which leaf is the FFEL. When considering the Rel_Abs_ and Abs_40_ (Fig. 4A,B) in Experiment 3, which are the results that would be used for heat tolerance and resistance interpretation, it was observed that the main stem leaf occurring just below the FFEL at approximately 16 days from unfolding provides results with no difference to those of the FFEL. While it is important to conduct an experiment with consistency in sampling, and the FFEL is still the ideal leaf for leaf disc sampling, when required, the main stem leaf below the FFEL can be a substitute without changing the assay results.

Future research is needed to translate these results to field conditions. Under the controlled glasshouse conditions of the experimentation presented in our study, it is expected that results would be consistent across any potential acclimation effects. Two aspects related to leaf age that require further experimentation in the context of the HSA are: (1) the effect of changes in cellular expansion and thus the number of cells sampled in a leaf disc, and (2) the possible effects of plant adaptation to imposed environmental conditions. When considering the possible effects of plant adaptation on the HSA, plant and/or genotype responses to environmental conditions may alter the results of the HSA. These responses include any biochemical or physiological response that may alter cellular expansion or viability, such as osmoregulation and turgor pressure^31–33^, synthesis of heat shock proteins to protect biochemical pathways^34,35^ and membrane integrity under abiotic stress^36^. These potential effects are particularly important when considering the dynamic growing environment of field-grown plants. While studies have shown consistent genotype differences in heat tolerance, regardless of exposure to high temperatures^15,37^, future studies should investigate the possible effects of genotype-by-environment interactions that may result in genotype rank changes in HSA performance, particularly given these studies are limited to two genotypes.

Experimental consistency is important as leaf age is proportional to cellular expansion^38,39^. This is important as the HSA is based on quantifying the viable cells within a leaf disc, where more formazan product is produced when more viable cells are present in a sample. Therefore, when collecting the relatively small 40mm^2^ leaf disc for analysis, differences in cellular expansion and thus the total number of cells sampled in a leaf disc may alter the results of the HSA. While Experiment 1 showed that the scaling down of leaf disc sample sizes does not affect the results of the HSA, a change in the total number of cells sampled in a leaf disc in response to leaf age may alter the HSA results. This trend was observed in Fig. 4, where maximum absorbance levels were observed in older, fully expanded leaves. Further work may be conducted assessing associations between HSA results and the number of cells within a leaf disc sample. Besides, as cellular expansion is one of the most sensitive processes to water deficit in cotton^40–42^, it is expected that inconsistencies in plant water deficit due to field variability or genotype response to water stress may also alter the results of the HSA.

In the field, heat stress can be exacerbated by and is often observed in tandem with drought stress. While the Australian cotton industry is characterised by fully irrigated conditions where this effect may be observed less frequently, it is still important to ensure that the plants sampled are not water-stressed when using the HSA. Both Abs_25_ and Abs_40_ were reduced by water stress. Thus, thus the sampling of water-stressed plants could potentially confound the results of the assay. This effect is most likely due to biochemical changes in the plant resulting from the effects of water stress treatment. Differences in cellular expansion in response to water stress could also influence the results. However, given the short duration of the water stress imposed, combined with our findings that leaf age/cellular expansion only has a minor effect on HSA, it is more probable that biochemical changes or loss of cellular viability impact the results. Passive rehydration of the leaf tissue (leaf discs were submerged in water immediately following collection) did not seem to ameliorate the impact of water stress. To identify heat tolerance, it is therefore important to ensure that variations in absorbance values from the HSA represent heat tolerance and resistance, and not water deficiencies, so plants should be kept well-watered. Finally, as responses to water stress vary among genotypes^43^ and this study was only conducted in one genotype, further studies investigating the probable presence of genotype rank changes when samples are collected under water stress conditions should be conducted.

When conducting the HSA, it is also important to consider transport and cooled storage between the time leaf disc samples are collected and the start of laboratory steps (Experiment 5). As the HSA is based on the detection of cellular viability, cellular death before laboratory analysis would alter results. Thus, samples have historically been submerged in water and placed in cool storage immediately following collection and during transport to the laboratory. We were able to determine that provided samples are kept submerged in water within their 1.1 ml ELISA tubes at approximately 16 °C, Rel_Abs_, Abs_25_, and Abs_40_ results are unaffected by storage between 1 and 16 h. It is speculated that these results were observed as the hydration of the leaf tissue and cool storage maintained cellular viability and minimised cellular death. This finding provides flexibility when using the HSA, enabling collection and transport of leaf disc samples at field sites that may not be near the laboratory facilities for processing and analysing samples. Additionally, it suggests that large differences in time of sampling between samples in an experiment would not affect the Rel_Abs_, Abs_25_, and Abs_40_ values, as long as they are kept in cooled storage. This is particularly important when screening germplasm from large experiments.

When considering the duration of leaf tissue submersion in TTC (Experiment 6), the ideal duration was found to be between 10 and 18 h as this was when the TTC reaction was no longer occurring and therefore, the absorbance values were no longer changing over time. Results of Run 1 for each absorbance measurement (Fig. 5A,C) showed changes based on the duration of the exposure to TTC, where the gap between 7 and 20 ho appeared to be a critical time. This was particularly evident in the Abs_25_ data as an absorbance peak was observed at 20 h, where only a trend in increasing absorbance values was observed in Abs_40_ data following 1 h of exposure to TTC. This shorter interval between the observation of maximum absorbance readings across stressed and non-stress conditions is expected as the duration of the TTC reaction would be shorter in situations where leaf tissue has reduced viability, i.e. the 40 °C treatment. When Run 2 was completed, all absorbance values indicated no difference between 10 and 18 h (Fig. 5B,D). This result suggests that anytime between 10 and 18 h would be acceptable for the duration of tissue submersion in TTC, assuming that within one experiment the duration chosen is kept consistent. However, due to possible variations in leaf morphology across genotypes such as waxiness, pubescence, and thickness that may alter TTC uptake and reaction times, we suggest selecting 16 h.

The approach to determining the optimal duration leaf tissue should be submersed in ethanol to extract the red formazan product for spectrophotometric analysis is different from that of the TTC submersion. In Experiment 7, the maximum absorption levels would suggest the ideal duration, as this would be before any potential degradation of the red formazan product. In each of the absorbance measurements, this peak was identified at approximately 13 h (Fig. 6A–C).

**Figure 7:**
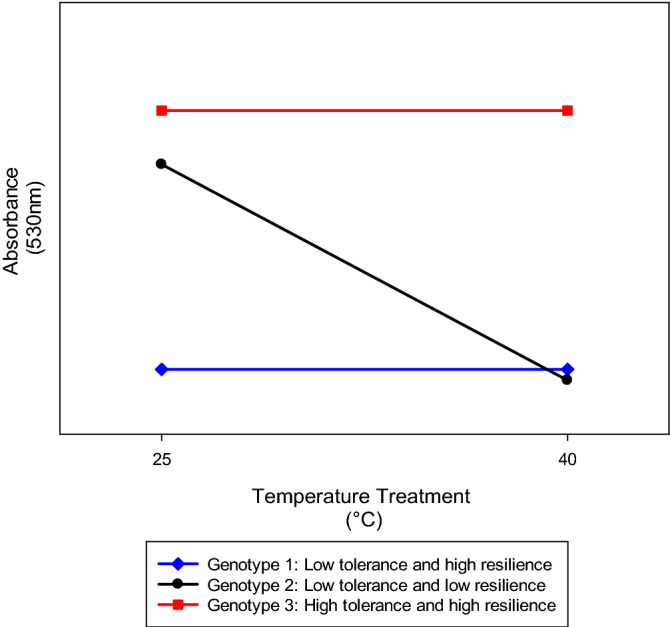
Theoretical outcomes of absorbance values for three genotypes when leaf material is non-stressed and heat-stressed (incubated at 25 and 40 °C, respectively) indicating differing levels of heat tolerance (absorbance at 40 °C) and resistance (slope).

In addition to refining the HSA method to provide a new level of confidence in the results obtained and ensuring the applicability of the assay to a breeding program, this study has also developed thinking around the interpretation of HSA results. For application of the HSA as an early-season screening method for breeding purposes, the results of this study suggest interpreting the results of the HSA in terms of both genotype heat tolerance and resistance. In this study, we define heat tolerance as a plant’s ability to maintain productivity or function following exposure to a heat stress event (i.e., the absolute value). In contrast, heat resistance is determined by the lack of change in productivity or function between non-stressed conditions and exposure to a heat stress event (i.e. the degree of difference between non-stressed and heat-stressed conditions). In the context of the HSA, heat tolerance can be described by the absorbance reading measurement following exposure to a heat stress event, i.e., 40 °C treatment, Abs_40_. However, because leaf tissue is exposed to non-stressed and elevated temperatures in a water bath prior to the addition of TTC solution, resistance is determined by the lack of change in absorbance between non-stressed conditions and exposure to a heat stress event (Rel_Abs_, or slope of the line between Abs_25_ and Abs_40_). Figure 7 shows theoretical outcomes of absorbance values for three cotton genotypes when leaf material is non-stressed (incubated at 25 °C) and heat-stressed (incubated at 40 °C). As shown, Genotype 1 has a relatively low absorbance value at both 25 °C and 40 °C. Because the value is low at 40 °C, the genotype is considered to have low heat tolerance. However, because there was no change between the absorbance at 25 °C and 40 °C, this genotype is considered heat resistant with similar performance regardless of the temperature treatment. Similarly, Genotype 2 has a low absorbance value at 40 °C and therefore has low heat tolerance. The difference is, in Genotype 2, the absorbance value at 40 °C has dramatically decreased from the reading at 25 °C (slope) and therefore, this genotype exhibits low resistance. Lastly, Genotype 3 shows both high heat tolerance and resistance. For breeding purposes, these results can be used at the discretion of the breeder. If high heat tolerance is desired, the breeder can place more selection pressure on spectrophotometric values from tissue exposed to a heat stress event. However, if consistent performance is expected under both stressed and non-stressed temperature conditions, the breeder can look to the resistance outcomes, as well as any combination of both heat tolerance and resistance.

## Conclusions

In this paper, a refined HSA methodology has been developed as a standard small-scale methodology utilizing TTC as a screening method for identifying and differentiating vegetative heat-tolerant genotypes of cotton. The HSA is advantageous as it provides a useful method for the identification of a physiological pathway of heat tolerance, cellular viability under high-temperature stress. The HSA provides a reliable methodology that can be tailored to the breeder's interests based on heat tolerance and/or resistance. Future research should be conducted where the HSA is used to identify individuals in a segregating breeding population with high cellular viability following heat stress. These individuals must then be tested under field conditions where heat stress is imposed, assessing the impact of improved cellular viability under heat stress on crop yield. Furthermore, while the HSA targets vegetative heat tolerance, multiple heat tolerance mechanisms at various growth stages may be required to maintain crop productivity under high-temperatue conditions. Thus, vegetative heat tolerance, as measured by the HSA or other techniques such as cell membrane thermostability or chlorophyll fluorescence, may not always correlate with reproductive-stage thermotolerance. Thus, a combination of vegetative and reproductive heat tolerance mechanisms, such as pollen viability and fruit retention, may be required to confer field thermotolerance with respect to crop yield. Future research should focus on not just the field performance of material selected using the HSA, but also the development of reproductive termotolerance. This may include the use of TTC based cell viability assays in pollen exposed to different thermal environments.

The development of methodologies to screen germplasm for physiological heat tolerance is important. This is because (1) physiological traits are genetically complex and dynamic with respect to time and the degree of stress imposed; and (2) the presence of negative associations with yield results in difficulties in capturing germplasm with physiological heat tolerance when selection is solely based on yield performance. Concurrent selection of yield and physiological heat tolerance, using methodologies such as the described heat stress assay, is expected to provide additional benefit when breeding germplasm for improved heat tolerance. While the concurrent selection of multiple, complex traits with potential negative associations has its challenges, the minimum requirement of a selection program is the accurate identification of phenotypes. This paper addresses this requirement by outlining a screening method for identifying heat tolerant phenotypes in cotton.

## Data Availability

The datasets used and/or analysed during the current study are available from the corresponding author on reasonable request.

## Abbreviations

Abs_25_: Spectrophotometric absorbance at 530 nm from 25 °C treated leaf disc
Abs_40_: Spectrophotometric absorbance at 530 nm from 40 °C treated leaf disc
FFEL: First fully expanded leaf
HSA: Heat stress assay
Rel_Abs_: Relative absorbance, i.e., the quotient of Abs_40_ and Abs_25_
SEM: Standard error of mean
TTC: Triphenyl tetrazolium chloride

## Research involving plants

The use of the plant material in this study, *Gossypium hirsutum* L. cv. Sicot 71, complies with all relevant institutional, national, and international guidelines and legislation.
